# Chemical Recycling of Used PET by Glycolysis Using Niobia-Based Catalysts

**DOI:** 10.1021/acsengineeringau.2c00029

**Published:** 2023-01-03

**Authors:** Shadi Shirazimoghaddam, Ihsan Amin, Jimmy A Faria Albanese, N. Raveendran Shiju

**Affiliations:** † Van’t Hoff Institute for Molecular Sciences, University of Amsterdam, 1090 GDAmsterdam, The Netherlands; ‡ Catalytic Processes and Materials Group, Faculty of Science and Technology, MESA+ Institute for Nanotechnology, University of Twente, P.O. Box 217, 7500 AEAmsterdam, Netherlands

**Keywords:** plastics recycling, polymers, chemical recycling, PET, Niobia, heterogeneous catalysis, glycolysis

## Abstract

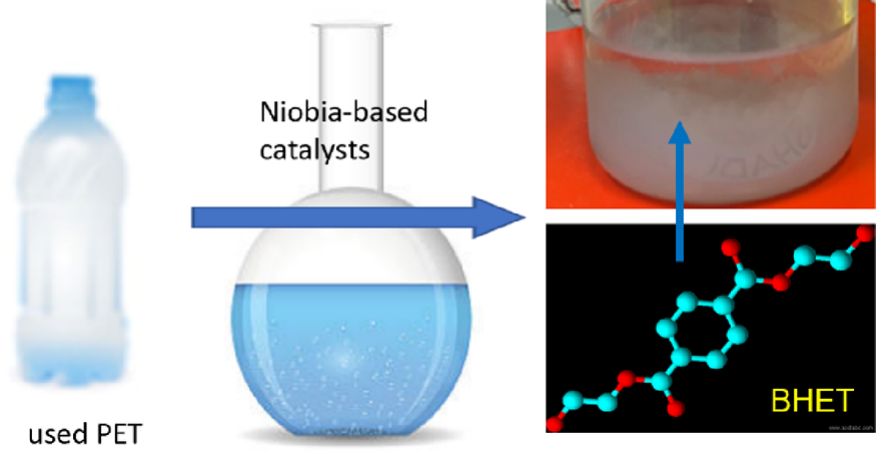

Plastic production has steadily increased worldwide at a staggering pace. The polymer industry is, unfortunately, C-intensive, and accumulation of plastics in the environment has become a major issue. Plastic waste valorization into fresh monomers for production of virgin plastics can reduce both the consumption of fossil feedstocks and the environmental pollution, making the plastic economy more sustainable. Recently, the chemical recycling of plastics has been studied as an innovative solution to achieve a fully sustainable cycle. In this way, plastics are depolymerized to their monomers or/and oligomers appropriate for repolymerization, closing the loop. In this work, PET was depolymerized to its bis(2-hydroxyethyl) terephthalate (BHET) monomer via glycolysis, using ethylene glycol (EG) in the presence of niobia-based catalysts. Using a sulfated niobia catalyst treated at 573 K, we obtained 100% conversion of PET and 85% yield toward BHET at 195 °C in 220 min. This approach allows recycling of the PET at reasonable conditions using an inexpensive and nontoxic material as a catalyst.

## 1 Introduction

Polyethylene terephthalate (PET) is a semicrystalline thermoplastic polyester with excellent water resistance, transparency, and mechanical strength produced by the polycondensation reaction of terephthalic acid and ethylene glycol.^1−3^ PET, due to its sturdy physicochemical and mechanical properties, low cost, and safe consumer use, has been widely used over the past decades in various applications and has become ubiquitous in our daily life. For instance, it can be used in the production of textile fibers, bioriented films, and packaging.^1,2,4^ The durability of PET, its mass production, and poor end-of-life management, unfortunately, have caused a serious global environmental problem.^4^

Approximately, 73 million tons of PET were produced globally in 2020, whereas only 9% of PET was recycled.^5^ Most of these disposable plastics ended up as trash accumulated in landfills or in the oceans, causing significant harm to the environment in terms of water, air, and land pollution. The decomposition of PET can take several decades; so, it is not surprising that biomagnification and bioaccumulation in oceans and other water bodies have transferred these materials to humans.^6^ Recently, researchers at the Free University of Amsterdam reported the presence of micrometer-sized plastic particles, including PET, in blood samples of healthy individuals.^7^ Similar results were reported by researchers at the Hull University on lung tissues.^8^ Clearly, immediate action is vital to tackle this issue before it becomes global public health and environmental problems.^9^ Also, PET plastics are produced from fossil-based raw materials, which increases the C intensity of the process. Therefore, developing technologies that can bring these materials back to the production cycle can reduce both the carbon footprint and the environmental pollution.^10^ If one ton of plastic waste is recycled,^10^ then seven barrels of oil could be saved; therefore, developing an efficient recycling strategy will reduce the energy and crude oil consumption.

Nonetheless, due to the economic and ecological concerns, depolymerization of PET has received scientists’ attention in the past years, which has led to significant attempts to recycle PET globally.^4^ Mechanical recycling is one of the options but restricted to few cycles as the mechanical and physicochemical properties of the plastic degrade over time, ultimately limiting the economical value of the finished product. Chemical recycling allows for the complete recovery of the individual monomers employed in the manufacturing of PET, which facilitates the production of pristine plastic. As the price of pristine PET continues to be the same, the recycling technologies should be economical to produce cheaper PET, which can profit the industries.^2^

There are four different mechanical and chemical recycling methodologies of PET substances: Primary recycling is the reusing of preconsumed PET materials that are uncontaminated and clean; for instance, preconsumed PET bottles can be used to produce new PET bottles.^2^ For secondary recycling, in this process, postconsumed PET materials undergo mechanical recycling, which includes different steps, the removal of contamination, drying, and melt reprocessing.^11^ Tertiary recycling or chemical recycling includes depolymerization of PET materials to their monomer units. Chemical recycling can be achieved through pyrolysis or by solvolysis.^2^ The pyrolysis process includes the depolymerization of PET materials by heat and in the absence of oxygen. In contrast, solvolysis is a process whereby PET material is depolymerized by solvents such as water (hydrolysis), alcohols (alcoholysis), amines (aminolysis), ammonia (ammonolysis), and glycol (glycolysis).^2,3^ It has to be noted that PET materials are a type of polyester, which contain ester functional groups that can be split by different reagents.^3^ For quaternary recycling, in this process, postconsumed PET materials get incinerated, and the produced energy content can be recovered. To illustrate this, wasted plastics, together with other municipal wastes, can get burned in advanced incinerators, and as a result, significant heat and steam will be generated, which can run the turbine blades and produce electricity.

**Figure 1:**
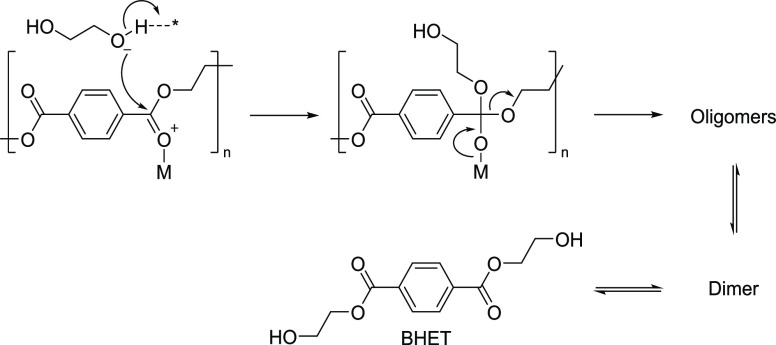
Representation of various stages of PET glycolysis using ethylene glycol.

Glycolysis, due to its advantages in terms of operating temperature, mild reaction conditions, yield, and scalability, is one of the most promising depolymerization processes for the chemical recycling of PET.^3,12−34^ The obtained monomers, which are bis(2-hydroxyethyl) terephthalate (BHET) and ethylene glycol (EG), have diverse applications.^35^ Among the different recycling processes,^36−40^ we have chosen glycolysis to convert the PET into its monomers (Figure 1).

PET glycolysis can be carried out using diols, such as ethylene glycol (EG), propylene glycol (PG), and diethylene glycol (DG). In the absence of catalysts, the reaction is slow, leading to incomplete depolymerization of PET to BHET monomers. Instead, the formation of side products (oligomers) is significantly enhanced. The low reactivity of PET is associated to the high stability of the carboxylic group as electron delocalization is enhanced by the resonance with the aromatic ring. For this reason, solid Brønsted acid catalysts are required to activate the carboxyl group and facilitate the electrophilic attack of EG to C=O.^2^

The advantages of solid acid catalysts outweigh the homogeneous acid catalysts, as it is possible to separate them from the reaction products by filtration, and consequently, the production of the hazardous waste will be lower. Also, solid acid catalysts are noncorrosive. Thus, the usage of solid acid catalysts in most industrial chemical reactions, as environmentally friendly catalysts, has been prevalent over the past decades.^3^

EG has a free electron pair that can start the reaction by attacking the carbonyl carbon of the ester group of PET.^41^ Consequently, the hydroxyethyl group of EG forms a bond with the carbonyl carbon of PET (Figure 1), resulting in splitting the long-chain into short-chain oligomers and ultimately BHET.^41^ Nonetheless, the rate of glycolysis of PET depends on variables such as the temperature, PET/EG ratio, time, and PET/catalyst ratio. For example, it has been reported that glycolysis of PET with a molar ratio of 1:6 (PET:EG) at temperatures ranging within 180–195 °C under a reflux condenser has the highest efficiency.^42^ Currently, the solid catalysts either require a high temperature or pressure to get a high yield. For example, ZnMn_2_O_4_ is reported to be one of the best catalysts, yielding 92% BHET yield, but it requires 260 °C and 5.0 atm pressure.^14^ Another solid catalyst, γ-Fe_2_O_3_ yielded >90% BHET but required a temperature of 300 °C.^17^ Moreover, for an economical operation, it is also required that the catalysts should be inexpensive and readily preparable and should have high mechanical and thermal stability.

We studied depolymerization/glycolysis of PET materials using sulfated niobia, a solid acid catalyst (Figure 1). Niobia is also a water-tolerant metal oxide. The calcination temperature of the catalysts and depolymerization conditions have been optimized to get the highest yield of BHET formation. This manuscript reports the results from these studies.

## 2 Materials and Methods

### 2.1 Materials

Nb_2_O_5_·*n*H_2_O, commercially known as HY-340, was granted by the Brazilian Company of Metallurgy and Mining (CBMM). A used PET water bottle was employed as a substrate. The bottle was washed and dried for 12 h and then cut into small pieces (1 mm × 1 mm). Ethylene glycol (EG) and ammonium sulfate were purchased from VWR Chemicals and Fluka, respectively.

### 2.2 Preparation of Catalysts for the Glycolysis of PET

For SO_4_^2–^/Nb_2_O_5_·*n*H_2_O, 15 mL of the solution of 0.5 M (NH_4_)_2_SO_4_ was prepared. Then, 1 g of niobium oxide was added to the solution at 80 °C under reflux while vigorously stirring for 3 h. Then, the mixture was filtered using a 0.45 μm size filter and dried at 110 °C for 16 h. After that, the powder was pulverized, and a portion was calcined at 300, 500, and 700 °C for 2 h.

### 2.3 Characterization Methods

Powder X-ray diffraction (XRD) analyses were carried out on a Rigaku MiniFlex II diffractometer using Cu Kα radiation (X-ray tube set at 30 kV and 15 mA). The XRD patterns were recorded between 2θ = 5–90° at a speed of 2.5°·min^–1^. A Thermo Scientific Surfer instrument was used to carry out N_2_ adsorption–desorption analyses at 77 K. Surface areas were determined with the Brunauer–Emmett–Teller (BET) method, and the mesoporosity was analyzed using the Barrett, Joyner, and Halenda (BJH) method. NMR spectra were recorded on a Bruker AMX 400 (400.1 and 100.6 MHz for ^1^H and ^13^C, respectively). The molecular weight of monomers/oligomers was determined by high-performance gel permeation chromatography (GPC). A refractive index detector was used for monitoring the products. G6000PWXL, G5000PWXL, and G3000PWXL columns were tandemly linked for analysis. Commercial dextran in the range of 5.22–2990 kDa was used to draw the calibration curve. The molecular weight of the analyte was calculated by the retention time. Fourier transform infrared spectroscopy (FTIR) was recorded in the region of 1400–500 cm^–1^, with a resolution of 4 cm^–1^ and 16 scans to identify the sulfate groups, using a PerkinElmer spectrophotometer Frontier Single Range-MIR.

### 2.4 Reaction Studies

PET (1 mm × 1 mm; 1 mol), 6 mol of ethylene glycol, and different catalysts were loaded into a 250 mL three-neck flask equipped with a magnetic stirrer, a thermometer, and a reflux condenser and heated in a sand bath. The reactions were carried out at temperatures ranging from 180 to 195 °C under atmospheric pressure for a certain time.

**Scheme 1:**
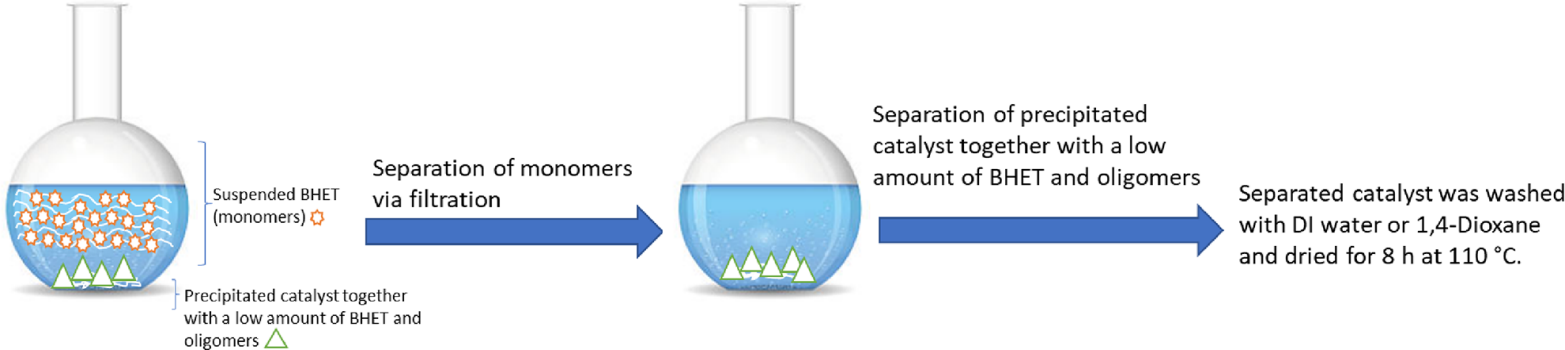
Separation of the BHET Monomer (yield), Unreacted PET, and the Catalyst after the First Cycle of Glycolysis

After the glycolysis reaction was finished, the reactor was cooled down to the ambient temperature. Undepolymerized PET was immediately collected and separated from the liquid phase and washed with distilled water. Then, the PET was dried and weighed. Meanwhile, 100 mL of distilled water was mixed with the water used to wash PET and was added to the reactor’s liquid phase while vigorously stirring at 70 °C for 30 min; this would dissolve the remaining BHET and EG. The insoluble fraction in water was a mixture of the oligomers, which was filtered, collected, dried, and not studied further in this report. Then, the solution (filtrate) was kept in a cold room (at 5 °C) for 16 h. White crystalline BHET flakes were formed, which were separated and dried in an oven at 70 °C for 3 h, while the separated catalyst was washed with demiwater and dried for 8 h at 110 °C before reuse (Scheme 1).

The yield of BHET and the PET conversion were calculated based on eqs 1 and 2, respectively.^2,10^ In the equations below, *W*_PET,*i*_ and *W*_PET,*u*_ refer to the initial and ultimate weight of PET, respectively.
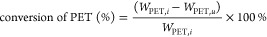
1
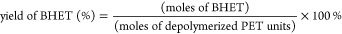
2

## 3 Results and Discussion

**Figure 2:**
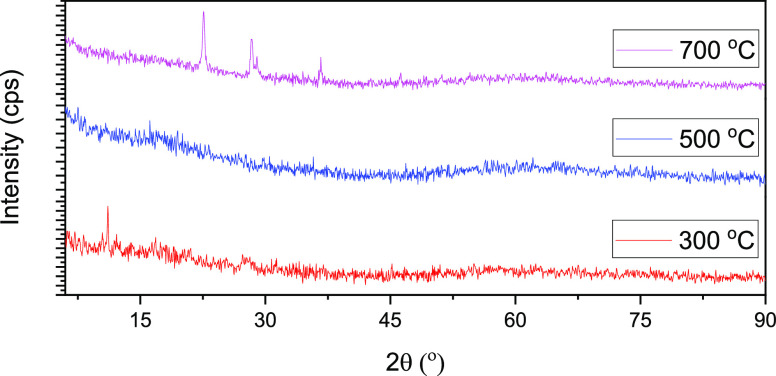
XRD patterns of SO_4_^2–^/Nb_2_O_5_·*n*H_2_O after calcination at 300, 500, and 700 °C.

Niobium-based catalysts prepared from commercial niobium oxide were characterized after calcination at 300, 500, and 700 °C for 2 h and further functionalization with sulfate groups. Figure 2 shows the XRD patterns of SO_4_^2–^/Nb_2_O_5_·*n*H_2_O-300 °C, SO_4_^2–^/Nb_2_O_5_·*n*H_2_O-500 °C, and SO_4_^2–^/Nb_2_O_5_·*n*H_2_O-700 °C. The catalysts calcined at 300 and 500 °C are in the amorphous state. The crystallization of hydrated niobium pentoxide Nb_2_O_5_·*n*H_2_O only occurs when it is calcined at a temperature above 500 °C as can be seen from the XRD peaks of SO_4_^2^/Nb_2_O_5_·*n*H_2_O-700 °C.^9,43^

Niobium pentoxide (Nb_2_O_5_) is the most thermodynamically stable polymorph in the niobium-oxygen system and can exist in the amorphous state or one of the different crystalline polymorphs depending on the treatment temperature.^43−46^ With elevating the calcination temperature, the material evolved into a more crystalline system. As the temperature of calcination increases, the surface is dehydroxylated. This favors the formation of more crystalline oxide phase.^3,47^ However, calcination at a lower temperature helps to maintain the Brønsted acidity on the surface of the catalyst.

**Figure 3:**
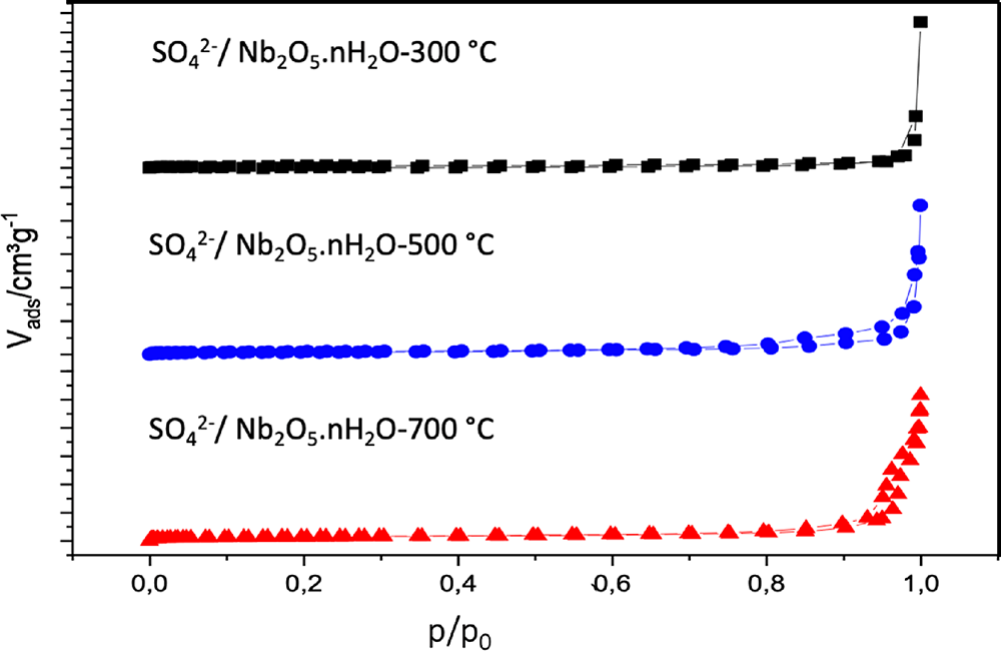
Nitrogen adsorption–desorption isotherms of SO_4_^2–^/Nb_2_O_5_·*n*H_2_O calcined at 300, 500, and 700 °C.

Figure 3 shows the nitrogen adsorption/desorption isotherms of the catalysts. The micropores of the catalyst get filled at a relatively low pressure (*P* < 0.01), and the quantity of the adsorbent depends on the amount of the micropores.^48^ However, the filling of the mesopores and macropores by the adsorbate molecules occurs at the relative pressure between 0.05 and 0.3.

Nitrogen physisorption indicates that our materials contain meso- and micropores as showcased by the hysteresis loop (see Figure 3). This is in line with the BJH analysis in which a significant fraction of mesopores are observed for all the materials. Surprisingly, increasing the calcination temperature led to higher surface areas. The isotherms can be considered type IV, where the hysteresis indicates mesoporosity. The volume of nitrogen (N_2_) adsorption increased at *P* > 0.95 because of the capillary condensation; subsequently, pore saturation occurred.^47^

Table 1 shows the surface areas of the catalysts. All catalysts have relatively small BET surface areas, and with the increasing calcination temperature, the BET surface area increases slightly. According to the BJH analysis, the SO_4_^2–^/Nb_2_O_5_·*n*H_2_O-300 °C catalyst contains mainly mesopores. Nonetheless, considering the small surface area, the pores might be occupied by sulfate groups. When the catalysts are calcined at elevated temperatures, these groups would decompose. We also prepared a catalyst with a lower amount of sulfate, which will be described in the following paragraphs. Table S1 shows the composition of SO_4_^2–^/Nb_2_O_5_·*n*H_2_O-300 °C obtained by micro-XRF, which shows that the amount of sulfate is about 36 wt %. This catalyst was used in the reactions initially.

**Table 1:** The Textural Properties of SO_4_^2–^/Nb_2_O_5_·*n*H_2_O Calcined at Different Temperatures

catalysts	BET surface area [m^2^ g^–1^]
SO_4_^2–^/Nb_2_O_5_·*n*H_2_O-300 °C	2.5
SO_4_^2–^/Nb_2_O_5_·*n*H_2_O-500 °C	6.4
SO_4_^2–^/Nb_2_O_5_·*n*H_2_O-700 °C	25

**Figure 4:**
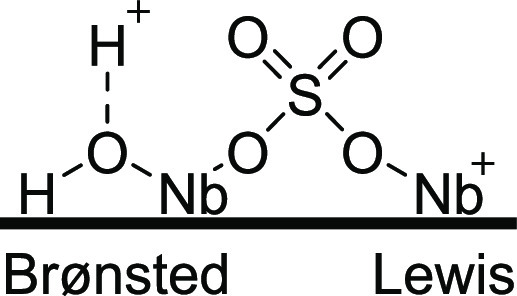
Illustration of Lewis and Brønsted acid sites on the surface of the SO_4_^2–^/Nb_2_O_5_·*n*H_2_O-300 °C.

Niobia has surface acidic sites due to the hydroxyl groups. The calcination at 300 °C does not significantly affect the acidity; however, calcination at elevated temperatures results in the elimination of the hydroxyls leading to a reduction in the acidity. The addition of sulfate groups enhances the Brønsted acidity of the catalyst (see Figure 4). Figure S1 shows the FTIR spectrum of the sample calcined at 300 °C.

#### 3.1.1 PET Glycolysis Reaction

**Figure 5:**
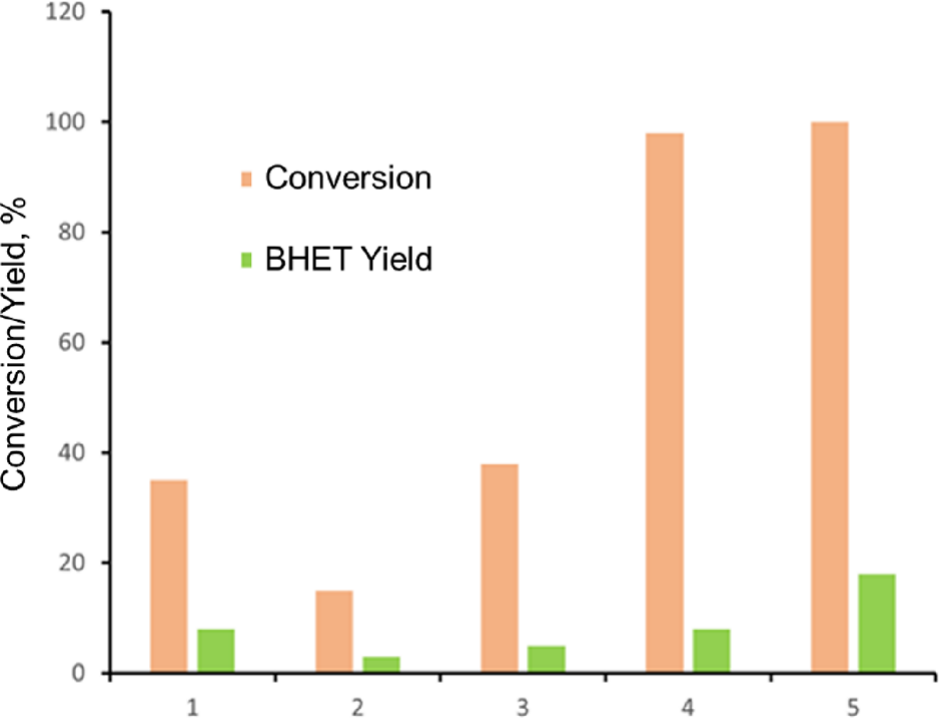
Glycolysis of PET with and without catalysts at 195 °C. (1) Without a catalyst, 360 min. (2) Phosphotungstic acid, 30 wt %, 360 min. (3) SO_4_^2–^/Nb_2_O_5_·*n*H_2_O-300 °C, 5 wt % catalyst loading, 180 min of reaction time. (4) SO_4_^2–^/Nb_2_O_5_·*n*H_2_O-300 °C, 10 wt %, 180 min. (5) SO_4_^2–^/Nb_2_O_5_·*n*H_2_O-300 °C, 20 wt %, 180 min. PET:EG mole ratio = 1:6.

Initially, the glycolysis of PET was carried out at 195 °C using SO_4_^2–^/Nb_2_O_5_·*n*H_2_O-300 °C as a catalyst with different catalyst loadings as shown in Figure 5. We also did reactions using phosphotungstic acid and without any catalyst at 195 °C for 6 h.

Phosphotungstic acid at these conditions acts as a homogeneous acid catalyst. Interestingly, the reaction without a catalyst had a higher yield than that of phosphotungstic acid. However, the yields in both cases were still insignificant compared to that of SO_4_^2–^/Nb_2_O_5_·*n*H_2_O-300 °C, and it took more time, 6 h, to depolymerize. These results show that the highest PET conversion and the yield toward the BHET were obtained for SO_4_^2–^/Nb_2_O_5_·*n*H_2_O-300 °C with a 20 wt % loading and a reaction time of 180 min. The presence of the sulfate group on the surface of the catalyst can boost the reactivity of the transesterification reaction and enhance the rate of the glycolysis reaction.^49^

### 3.2 Effect of Calcination Temperature and Time

**Figure 6:**
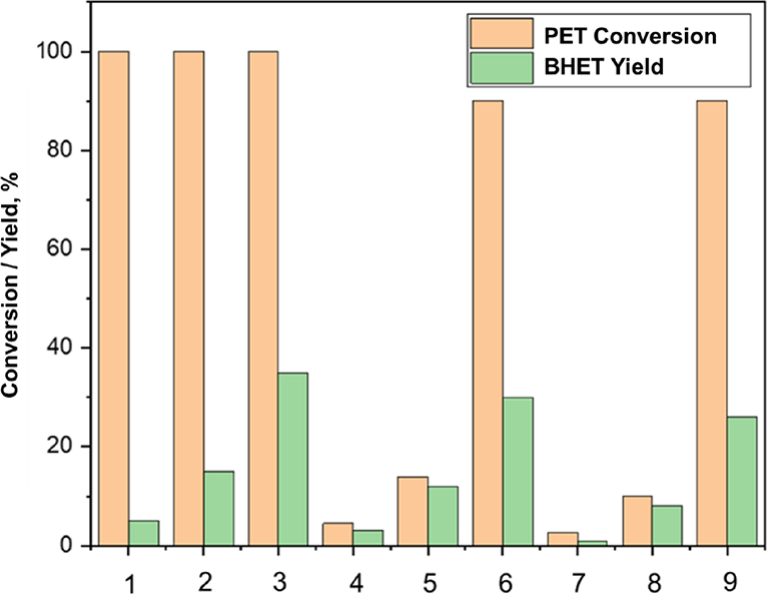
Effect of calcination temperature and reaction time on PET conversion and yield of BHET monomers at 195 °C. 20 wt % catalyst loading in all cases. (1) SO_4_^2–^/Nb_2_O_5_·*n*H_2_O-300 °C, 60 min of reaction time. (2) SO_4_^2–^/Nb_2_O_5_·*n*H_2_O–300 °C, 140 min. (3) SO_4_^2–^/Nb_2_O_5_·*n*H_2_O-300 °C, 220 min. (4) SO_4_^2–^/Nb_2_O_5_·*n*H_2_O-500 °C, 60 min. (5) SO_4_^2–^/Nb_2_O_5_·*n*H_2_O-500 °C, 140 min. (6) SO_4_^2–^/Nb_2_O_5_·*n*H_2_O-500 °C, 220 min. (7) SO_4_^2–^/Nb_2_O_5_·*n*H_2_O-700 °C, 60 min. (8) SO_4_^2–^/Nb_2_O_5_·*n*H_2_O-700 °C, 140 min. (9) SO_4_^2–^/Nb_2_O_5_·*n*H_2_O-700 °C, 220 min. PET:EG mole ratio = 1:6.

The calcination temperature has a significant impact on the structure of a solid acid catalyst and its properties. As shown in Figure 6, a lower PET conversion was observed for SO_4_^2–^/Nb_2_O_5_·*n*H_2_O-700 °C when compared to SO_4_^2–^/Nb_2_O_5_·*n*H_2_O-300 °C at the same mass loading. When the catalyst was calcined at a high temperature (700 °C), the hydroxyl groups on the catalyst surface were removed. The lower density of active sites could explain the lower conversion obtained with the catalyst calcined at 700 °C. Moreover, the yield of BHET monomers, when SO_4_^2–^/Nb_2_O_5_·*n*H_2_O-300 °C was used, was somewhat higher than those obtained with SO_4_^2–^/Nb_2_O_5_·*n*H_2_O-500–700 °C. Furthermore, when SO_4_^2–^/Nb_2_O_5_·*n*H_2_O-700 °C with a 20 wt % catalyst loading was used, oligomers with around 20% PET weight were produced. The conversion and yield to BHET increased with the reaction time irrespective of the calcination temperature of the catalysts.

Table 2 shows the results obtained when the loading of sulfated niobia calcined at 300 °C was increased to 40 wt %. When the reaction time increased to 220 min at this loading, a remarkable yield toward BHET was obtained. However, extended reaction times such as 8 h did not help as the yield of BHET decreased, which could be due to the polymerization of EG with monomers/dimers/trimers.

**Table 2:** The Effect of Time and the Catalyst Concentration on the Yield of BHET Monomers^a^

catalyst	reaction time (h)	conversion (%)	BHET yield (%)
SO_4_^2–^/Nb_2_O_5_·*n*H_2_O-300 °C 20 wt %	8	100	20 (brownish color)
SO_4_^2–^/Nb_2_O_5_·*n*H_2_O-300 °C 40 wt %	3.40 (220 min)	100	85
SO_4_^2–^/Nb_2_O_5_·*n*H_2_O-300 °C 40 wt %	8	100	26 (brownish color)

a Reaction temperature = 195 °C; PET:EG mole ratio = 1:6.2.

### 3.3 Effect of the EG Ratio

**Figure 7:**
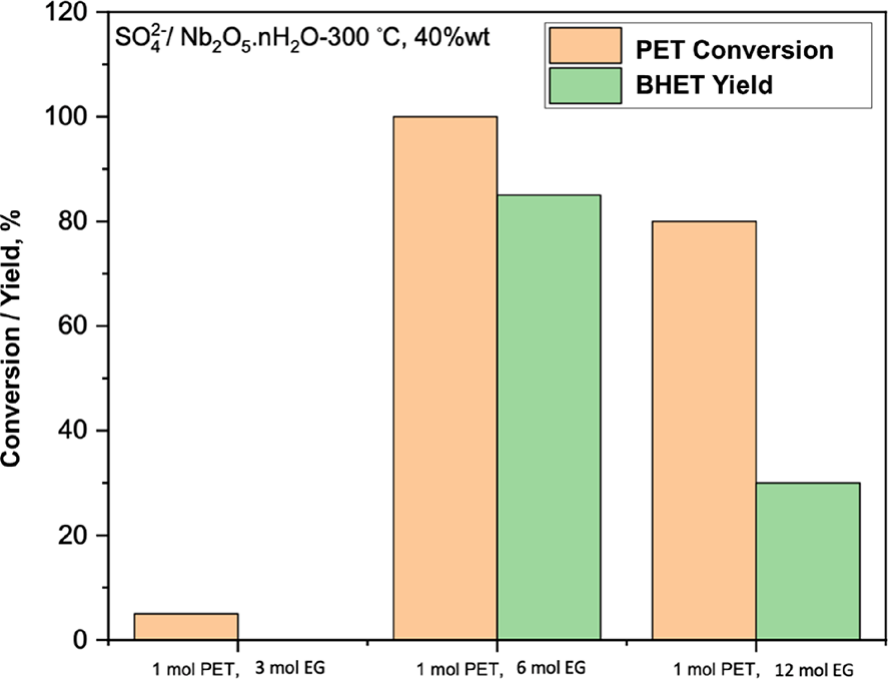
Effect of the amount of EG on PET glycolysis and BHET yield at 195 °C for 220 min with the utilization of SO_4_^2–^/Nb_2_O_5_·*n*H_2_O-300 °C; 40 wt %; PET:EG mole ratio = 1:6.

We also studied the effect of the amount of EG on PET glycolysis. In Figure 7, the results of different ratios of EG to PET are summarized. At 195 °C for 220 min and a 40 wt % catalyst weight, the 1:6 mole ratio of PET:EG gave 100% conversion of PET and 85% yield toward BHET. However, as the amount of EG increased, the PET conversion and yield of BHET decreased. This may be due to the lower chance of PET to contact catalyst active sites when the reaction mixture was diluted by an excess of EG.

### 3.4 Effect of the Catalyst Loading

Table 3 shows the effect of the catalyst loading. By increasing the catalyst concentration from 20 to 40 wt %, the yield toward BHET increased significantly, almost quadrupled. This could be due to the higher availability of the catalyst and the higher chance that PET materials come in contact with active sites on the catalyst’s surface. However, when the amount of the catalyst increased to 60 wt %, the conversion of PET and yield toward BHET decreased substantially. This could be due to the high solid content in the reaction mixture, which makes the functioning of EG not proper.

**Table 3:** The Effect of the Catalyst Loading on the BHET Yield^a^

catalyst	reaction time (min)	conversion (%)	yield toward BHET (%)
SO_4_^2–^/Nb_2_O_5_·*n*H_2_O-300 °C 20 wt %	220	100	20
SO_4_^2–^/Nb_2_O_5_·*n*H_2_O-300 °C 40 wt %	220	100	85
SO_4_^2–^/Nb_2_O_5_·*n*H_2_O-300 °C 60 wt %	220	10	10

a SO_4_^2–^/Nb_2_O_5_·*n*H_2_O-300 °C catalyst; 195 °C; 220 min.

### 3.5 Effect of the Catalyst Loading and Solvent Concentration on the Reaction

We did more experiments regarding the catalyst loading and EG concentration within a different time frame to optimize reaction conditions. As presented in Table 4, for SO_4_^2–^/Nb_2_O_5_·*n*H_2_O-500 °C, with the increasing EG concentration from 10:1 to 12:1 (EG:PET molar ratio), the yield toward BHET decreased slightly from 36 to 30%. Furthermore, when the amount of SO_4_^2–^/Nb_2_O_5_·*n*H_2_O-500 °C increased from 40 to 60%, the depolymerization yield dropped considerably; this is because the total amount of the solid reagent concentration is high, and glycolysis did not take place completely. A similar trend was found for SO_4_^2–^/Nb_2_O_5_·*n*H_2_O-700 °C. By extending the reaction time to 8 h, brownish products were formed due to the polymerization of BHET monomers in the presence of EG. Overall, by analyzing the results, we confirm that the SO_4_^2–^/Nb_2_O_5_·*n*H_2_O-300 °C catalyst has the highest BHET yield with optimized conditions of 40 wt % of the catalyst and a 6:1 mole ratio of EG:PET.

**Table 4:** The Effect of the Catalyst Loading and Solvent Concentration on Glycolysis of PET and BHET Yield

catalyst	catalyst loading (wt %)	EG:PET ratio (mol)	reaction time (min)	BHET yield (%)
SO_4_^2–^/Nb_2_O_5_·*n*H_2_O-300 °C	40	6:1	220	85
SO_4_^2–^/Nb_2_O_5_·*n*H_2_O-300 °C	40	10:1	220	61
SO_4_^2–^/Nb_2_O_5_·*n*H_2_O-500 °C	40	10:1	200	36
SO_4_^2–^/Nb_2_O_5_·*n*H_2_O-500 °C	40	12:1	220	30
SO_4_^2–^/Nb_2_O_5_·*n*H_2_O-700 °C	40	6:1	480	7
SO_4_^2–^/Nb_2_O_5_·*n*H_2_O-500 °C	60	10:1	160	30

### 3.6 Optimizing the SO_4_^2–^/Nb_2_O_5_·*n*H_2_O-300 °C Catalyst

We also changed the sulfate content on the catalyst. Preparation was done as before with a lower amount of (NH_4_)_2_SO_4_. Glycolysis of PET was performed by following the same procedure for 5 h. The catalyst gave 100% PET conversion and almost 60% yield for BHET (Table 5). This is 20% lower yield of BHET compared to that obtained with niobia impregnated with 40% (NH_4_)_2_SO_4_. As shown in Table 5, when the amount of impregnated sulfate is lower, the catalyst has a higher surface area because of the fewer ammonia and sulfate groups available to occupy the pores of the catalyst.

**Table 5:** Summary of the Results of Glycolysis of PET with the Usage of SO_4_^2–^/Nb_2_O_5_·*n*H_2_O-300 °C (20% (NH_4_)_2_SO_4_) and Its Textural Properties

catalyst	BET surface area [m^2^ g^–1^]	PET conversion (%)	BHET yield (%)
Nb_2_O_5_·*n*H_2_O	143		
SO_4_^2–^/Nb_2_O_5_·*n*H_2_O-300 °C (20% (NH_4_)_2_SO_4_)	97	100	60

**Figure 8:**
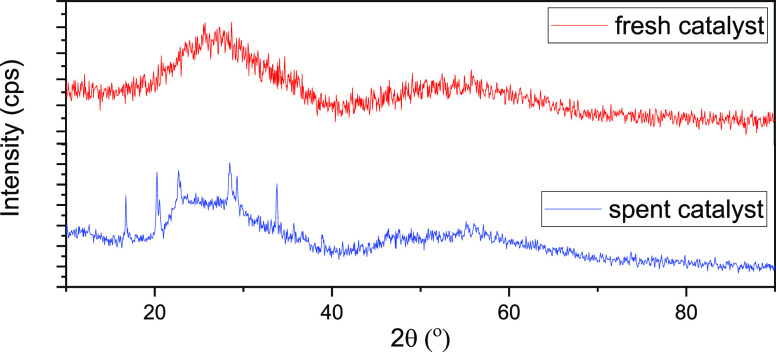
XRD patterns of SO_4_^2–^/Nb_2_O_5_·*n*H_2_O-300 °C (20% ammonium sulfate): (a) fresh catalyst (before usage) and (b) spent catalyst (after usage).

The recyclability and stability of SO_4_^2–^/Nb_2_O_5_·*n*H_2_O-300 °C with a 20% sulfate loading were studied. The catalyst was recyclable and was able to catalyze the reaction again. The catalyst after the first glycolysis reaction was separated simply by filtration and subsequently was washed with demi water and then dried at 110 °C for 8 h. Afterwards, the catalyst was pulverized and again was used in the second glycolysis reaction at the same conditions. When the catalyst is recovered from the reaction mixture, it still contained some BHET monomers, as indicated by the XRD patterns shown in Figure 8 (see also Figure S4). The peaks in the pattern of the spent catalyst originate from BHET. Therefore, we also conducted a washing with dioxane to recover all BHET and to purify the catalyst. This step ensured that all the product is recovered and the catalyst is regenerated.

## 4 Conclusions

In summary, we successfully depolymerized the PET from used water bottles into its monomers and oligomers utilizing inexpensive niobia-based catalysts. Niobium pentoxide is an eco-friendly, inexpensive, and easily available material. The yield of glycolysis of PET strongly depends on different variables such as the calcination temperature of the catalyst, reaction time, reaction temperature, and PET/EG ratio. Under the optimum conditions of 195 °C, 220 min, and a PET:EG weight ratio of 1:6, sulfated (40 wt %) niobia calcined at 300 °C gave 100% conversion of PET and 85% yield toward BHET. When 20 wt % sulfate was used, the yield toward BHET monomers was around 60%. The catalyst can be recycled, which makes it promising for the industrial application.

## Supporting Information Available

Additional experimental details, materials, and methods, including catalytic activity tests, FTIR, XRD, XRF, NMR, GPC, and nitrogen adsorption/desorption (PDF)
