# Supplementary material for: Chemical Recycling of Used PET by Glycolysis Using Niobia-Based Catalysts
Source: ACS Eng Au. 2023 Jan 3;3(1):37–44. doi: 10.1021/acsengineeringau.2c00029 (PMC9936547; doi:10.1021/acsengineeringau.2c00029)
Supplement: Supplementary file 1 — eg2c00029_si_001.pdf [file eg2c00029_si_001.pdf]

Supporting Information

Chemical Recycling of used PET by Glycolysis using Niobia-based Catalysts

Shadi Shirazimoghaddam,[1] Ihsan Amin,[1] Jimmy A Faria Albanese,[2] and N. Raveendran Shiju[1,*]

[1]Van 't Hoff Institute for Molecular Sciences, University of Amsterdam, 1090 GD Amsterdam, The Netherlands

[2]Catalytic Processes and Materials Group, Faculty of Science and Technology, MESA+ Institute for Nanotechnology, University of Twente, PO Box 217, 7500 AE, Netherlands

Catalytic activity tests. 1 mol of PET (1 mm x 1 mm), 6 mol of ethylene glycol, and different catalysts were loaded into a 250 ml three-neck flask equipped with a magnetic stirrer, a thermometer, a reflux condenser and heated in a sand bath. The reactions were carried out at temperatures ranging from 180 °C to 195 °C under atmospheric pressure for a certain time. It has to be noted that the different amount of catalyst has a different amount of metallic element which is correlated to the different catalytic activity[2].

After the glycolysis reaction was finished, the reactor was cooled down to the ambient temperature. Un-depolymerized PET was immediately collected and separated from the liquid phase and washed with distilled water. Then the PET was dried and weighed. Meanwhile, 100 ml of distilled water was mixed with the water used to wash PET and were added to the reactor's liquid phase while vigorously stirring at 70 °C for 30 min; this would dissolve the remaining BHET and EG. The insoluble fraction in water was a mixture of the oligomers, which was filtered, collected, dried, and not been studied further in this report. Then the solution (filtrate) was kept in a cold room (at 5 °C) for 16 h. White crystalline BHET flakes were formed, then separated and dried in an oven at 70 °C for 3 h. The yield of BHET and the PET conversion was calculated based on Eqs_1 and Eqs_2, respectively[10,2]. In the equations below, $W_{PET,i}$ and $W_{PET,u}$ refer to the initial and ultimate weight of PET, respectively.

$$Conversion\ of\ PET\ (\%) = \frac{(W_{PET,i} - W_{PET,u})}{W_{PET,i}} \times 100\% \quad (Eq.1)$$

$$Yield\ of\ BHET\ (\%) = \frac{(moles\ of\ BHET)}{(moles\ of\ depolymerized\ PET\ units)} \times 100\% \quad (Eq.2)$$

Acid analysis for the prepared catalyst. By studying the acidity of the catalysts with the Infrared method, it is possible to differentiate between Lewis and Brønsted acid sites. As is illustrated in Figure S1, which is the infrared spectrum of sulfated niobium pentoxide, several bands were observed at around 700 cm^{-1}, 900 cm^{-1}, 1100 cm^{-1}, 1450 cm^{-1}, and 3500 cm^{-1}. The IR bands at 1450 cm^{-1}, and a broad peak around 3500 cm^{-1} are attributed to Brønsted acid sites, whereas the IR bands at 700 cm^{-1}, 900 cm^{-1}, 1100 cm^{-1} are assigned to Lewis acid sites.

Furthermore, the infrared spectrum proved the presence of the sulfate functional group through a broad peak in the 1100-1470 cm^{-1}, which is mainly devoted to the symmetrical and asymmetric stretching of the S-O bands of SO_4^{2-} [3, 26].

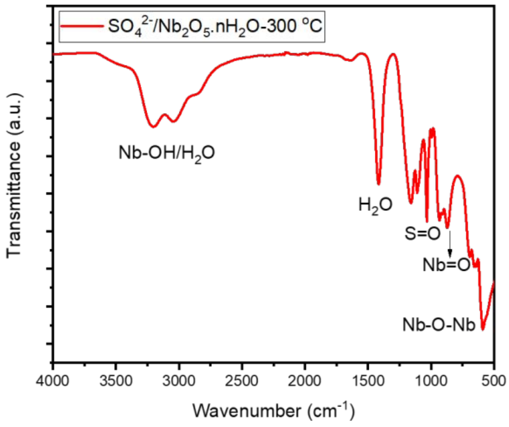

Figure S1. FTIR spectrum of $SO_4^{2-}/Nb_2O_5.nH_2O$-300°C.

Product Characterization. Confirmation of the final products was done by different characterization approaches such as FT-IR, H^{1}NMR and GPC. The IR spectrum of purified oligomer and BHET, which are the products of glycolytic depolymerization of PET, comprises –OH band at 3447 and 1136 cm^{-1}, C=O stretching at 1716 cm^{-1}, aromatic C–H at 1369 and 1505 cm^{-1}, and alkyl C–H at 2876-2961 cm^{-1}. It has to be noted that the hydroxyl peak of oligomers is insignificant since it has a longer chain and less hydroxyl group available. These results are analogous to the previous reports of glycolysis of PET bottles.[28, 29]

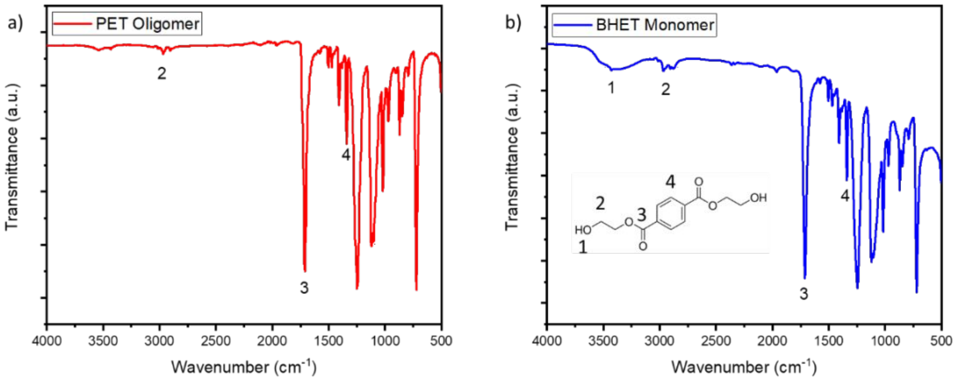

Figure S2. FT-IR spectra of oligomers and BHET.

Table S1. The results of XRF characterization of SO_4^{2-}/ $Nb_2O_5.nH_2O$-300 °C.

Formula	Weight (%)	Net int.	Stat. error (%)
Nb_2O_5	64	1305	0.1
SO_4^{2-}	36	16.5	0.8

The ^{1}H-NMR spectrum contains the peak at δ 8.2 ppm corresponds to the four aromatic ring protons. The signal at δ 1.6 indicates the presence of –OH groups, δ 3.96 and δ 4.52 correlate with an aliphatic (–CH_2) proton[26].

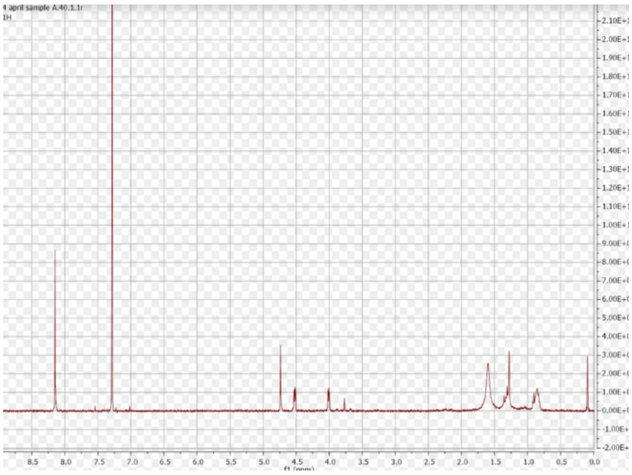

Figure S3. ^{1}H-NMR spectra of BHET contains the peak at δ 8.2 ppm corresponds to the four aromatic ring protons. The signal at δ 1.6 indicates the presence of –OH groups, δ 3.96 and δ 4.52 correlate with an aliphatic (–CH_2) protons.

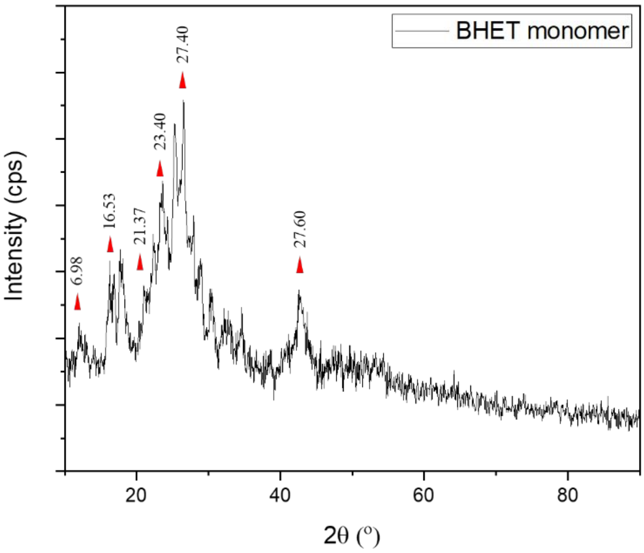

Figure S4. XRD pattern of BHET sample.

Figure S4 illustrates the XRD pattern of BHET monomers, showing the peaks at 2θ values of 6.98°, 16.53°, 21.37°, 23.40°, 27.40° and 42.60° representing the BHET monomers. The diffraction peaks of BHET are sharp with somewhat high intensity, which implies that monomers have high crystallinity.[30] Moreover, the GPC characterization was done to study the molecular weight of BHET monomers (MW=107 MW) and PET oligomers (MW=628), which can be found in the Supporting Information 1.2.

Supporting Information 1.1

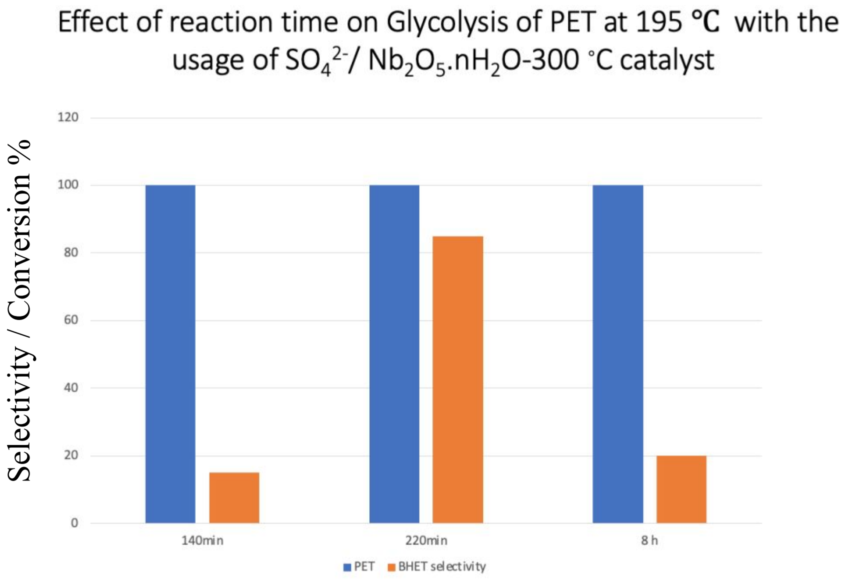

Supporting Information 1.2. GPC results of PET oligomer and BHET monomers.

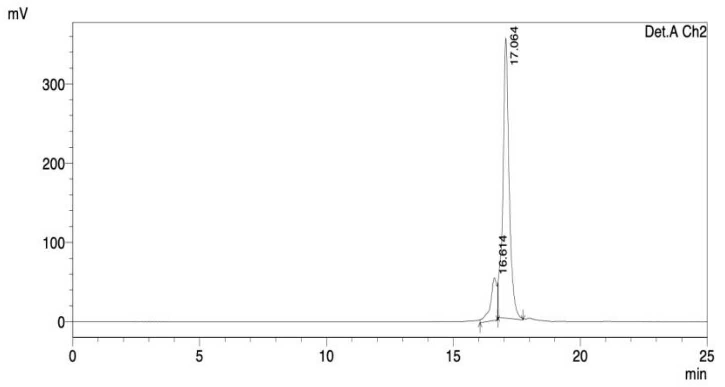

BHET (monomer) chromatogram.

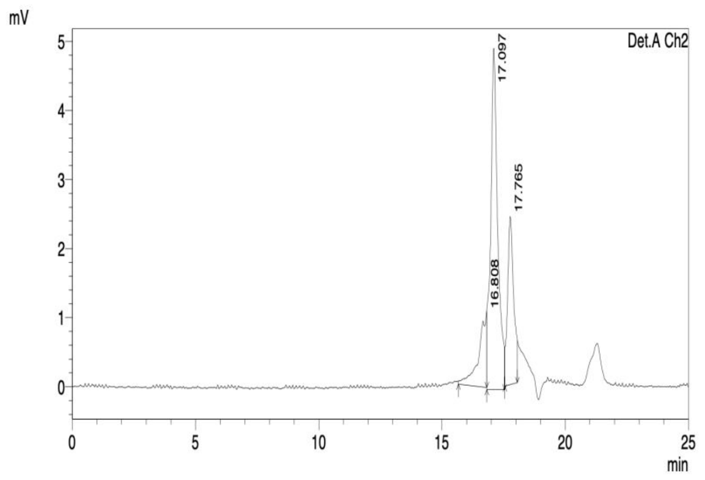

Oligomer chromatogram.

Supporting Information 1.3

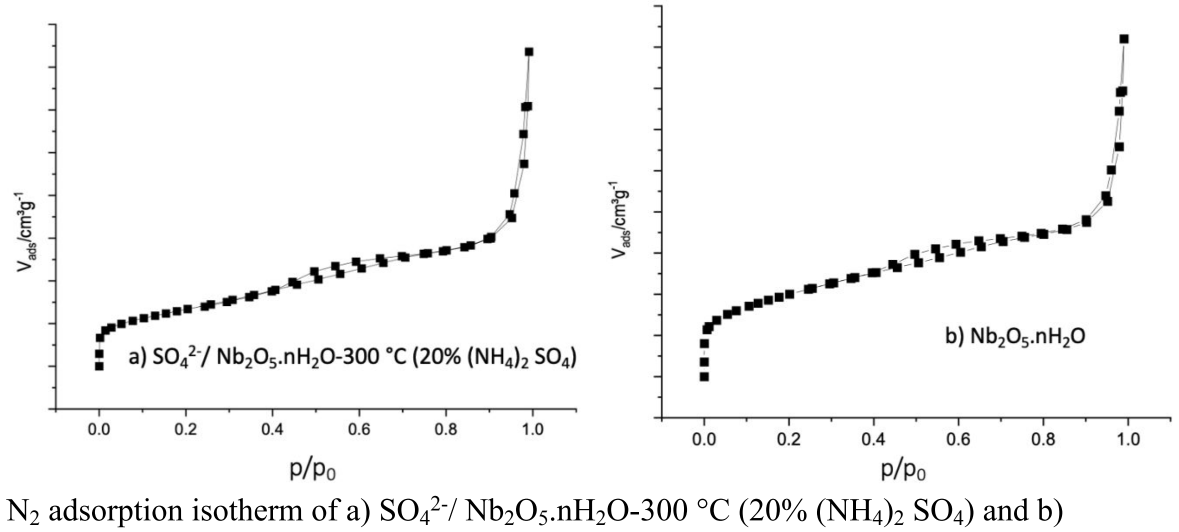

N_2 adsorption isotherm of a) SO_4^{2-}/ $Nb_2O_5.nH_2O$-300 °C (20% $(NH_4)_2$ SO_4) and b) $Nb_2O_5.nH_2O$

Supporting Information 1.4

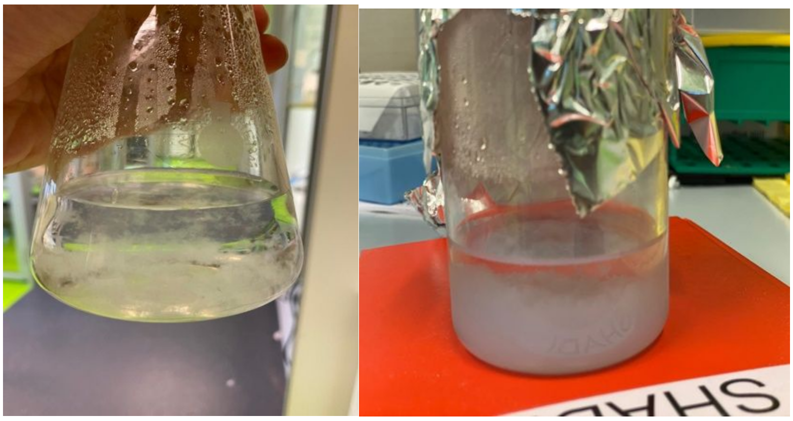

After glycolysis of PET and placing the filtrate in a cold room for 16 h, white crystalline BHET flakes were formed.
